# Angiotensin-Converting Enzyme (ACE) Inhibitor-Induced Angioedema in an African American Male With Coronavirus Disease 2019 (COVID-19)

**DOI:** 10.7759/cureus.60852

**Published:** 2024-05-22

**Authors:** Richard M Bresler, Jacob Whelan

**Affiliations:** 1 Internal Medicine, Baptist Memorial Hospital North Mississippi, Oxford, USA

**Keywords:** severe acute respiratory syndrome coronavirus 2 (sars-cov-2), ace inhibitor, angiotensin-converting enzyme 2 (ace-2), black or african american, bradykinin-induced angioedema

## Abstract

Angioedema is a condition characterized by non-pitting swelling of the subcutaneous or submucosal tissues in particular the face, lips, and oral cavity. Angiotensin-converting enzyme (ACE) inhibitors are known to contribute to the development of angioedema by increasing the levels of bradykinin and its active metabolites. Infection with severe acute respiratory syndrome coronavirus 2 (SARS-CoV-2) is hypothesized to contribute to the development of angioedema by modifying ACE II levels and further increasing the level of bradykinin in patients taking ACE inhibitors. African Americans may be at particular risk of developing angioedema with concomitant SARS-CoV-2 infection and ACE inhibitor use. This case involves a 31-year-old African American male diagnosed with coronavirus disease 2019 (COVID-19) who developed angioedema while taking an ACE inhibitor.

## Introduction

Angioedema is described as non-pitting edema of the subcutaneous and submucosal tissues [1]. It is caused by vasodilation and increased vascular permeability as a result of allergic and non-allergic triggers. Angiotensin-converting enzyme (ACE) inhibitors are a class of anti-hypertensive medications used by approximately 40 million people worldwide [2]. These medications have been associated with non-allergic angioedema in a small percentage of patients [3]. Severe acute respiratory syndrome coronavirus 2 (SARS-CoV-2), a member of the betacoronavirus family, spread worldwide in 2019. Endothelial dysfunction, increased vascular permeability, and systemic inflammation are well-documented virulent features of the infection [4]. This case describes a 31-year-old African American male who developed angioedema while taking an ACE inhibitor. He was found to be positive for coronavirus disease 2019 (COVID-19) upon admission. Infection with SARS-CoV-2 may have been a factor that contributed to the development of angioedema in this patient. African Americans, who have a genetic predisposition to developing angioedema independent of the aetiology, may be particularly susceptible to this complication. We hope this case will raise awareness of this clinical presentation and promote further investigation.

## Case presentation

**Figure 1:**
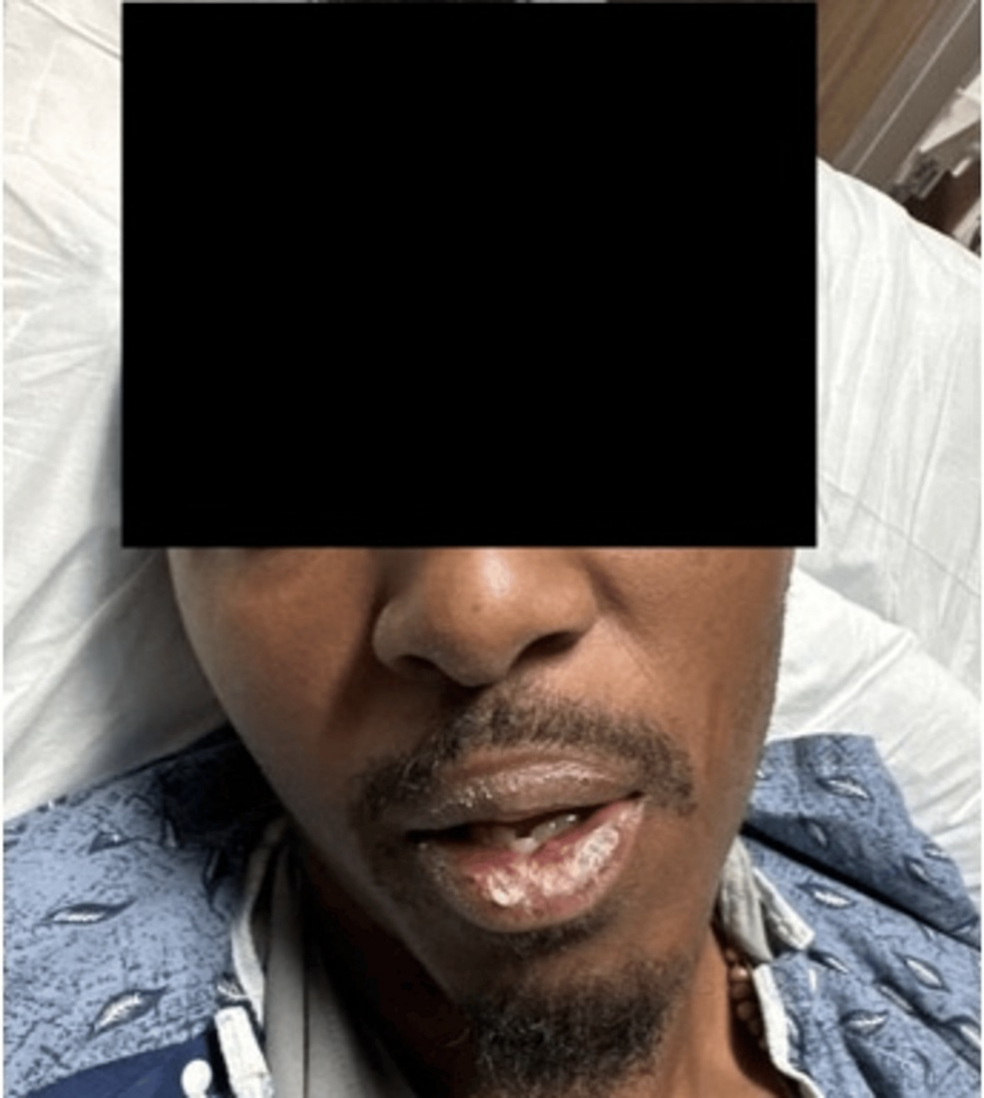
Pronounced, slightly asymmetric, non-pitting edema of the lips and face

A 31-year-old male with a past medical history of hypertension and asthma presented to the emergency department in spring with a two-day history of gradual lip, tongue, and facial swelling. He experienced shortness of breath, a dry cough, and body aches. He denied a rash or pruritis. The patient was started on lisinopril one month prior to admission for hypertension. He had no personal or family history of angioedema or face and tongue swelling. He denied prior asthma exacerbations or other respiratory conditions. Family history was significant for asthma in his son and daughter. He endorsed an anaphylactic reaction to shellfish as a child. The patient denied eating any shellfish-containing products recently or any foods outside of his regular diet prior to hospitalization. There was no recent exposure to insect bites, insect stings, antibiotics, or nonsteroidal anti-inflammatory drugs. He smoked half a pack of cigarettes daily for the past five years. The patient was up to date on all vaccinations and had received two COVID-19 vaccines. His vital signs on admission were the following: temperature of 36.5°C, heart rate of 93, blood pressure of 164/72, respiratory rate of 22, and room air oxygen saturation of 99%. On physical examination, he had difficulty swallowing without issues in talking or breathing. No respiratory distress was present. There was pronounced, slightly asymmetric non-pitting edema of the lips, tongue, and face (Figure 1).

**Figure 2:**
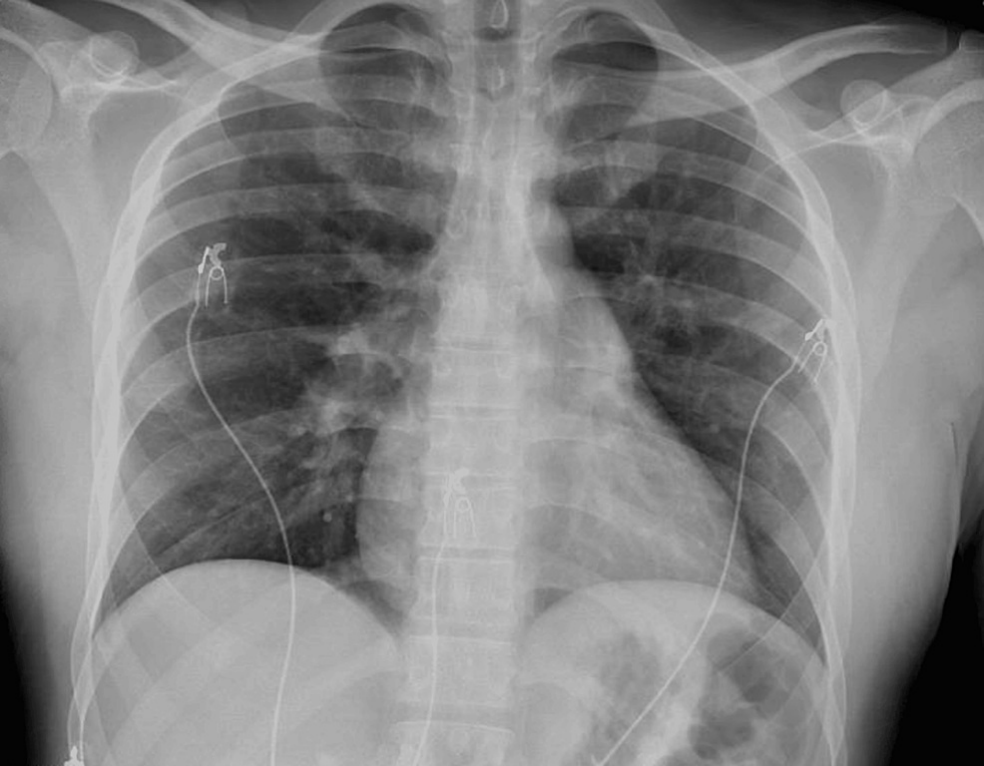
Chest X-ray showing no abnormalities

Auscultation of the lungs revealed decreased breath sounds bilaterally without any wheezes or rhonchi. A chest X-ray was unremarkable (Figure 2).

He had no other sites of swelling or urticarial eruption. Laboratory investigations revealed a white blood cell count of 6.0 K/uL, an eosinophil count of 0.01 K/uL, and a C4 level of 44 mg/dL (Table 1).

**Table 1:** Laboratory values

Laboratory tests (units)	Result	Reference range
White blood cells (K/uL)	6.0	5.0-10.0
Eosinophil count (K/uL)	0.01	0.05-0.50
C4 (mg/dL)	44	10.0-40.0

**Figure 3:**
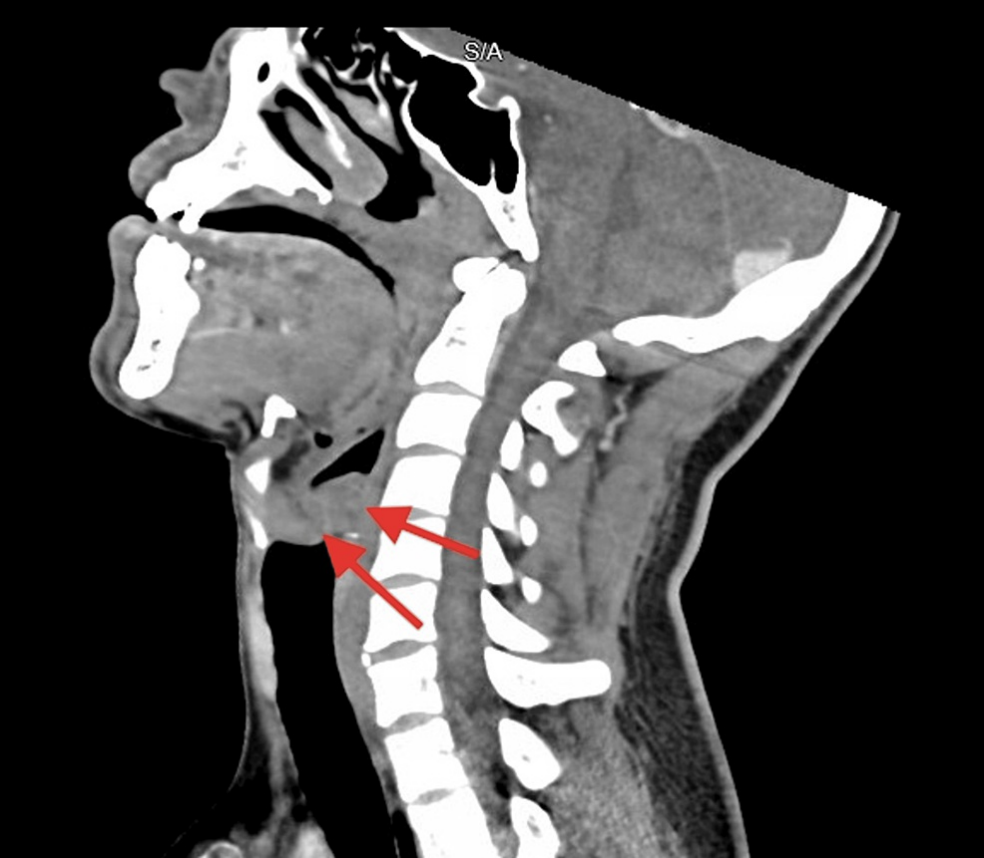
CT of the head and neck in a sagittal view showing mild thickening of the epiglottis (arrows)

SARS-CoV-2 infection was confirmed by a positive SARS-CoV-2 nasopharyngeal molecular swab. CT of the neck demonstrated mild thickening of the epiglottis concerning for acute epiglottitis (Figure 3).

A dose of intravenous ceftriaxone 1 g was administered, and fiberoptic laryngoscopy was performed at the bedside given the concern for acute epiglottitis on the CT scan. A normal-appearing epiglottis and patent airway were visualized. The patient was treated with intravenous Solu-Medrol 60 mg every eight hours, intravenous diphenhydramine 12.5 mg every six hours, and remdesivir for suspected ACE inhibitor-induced angioedema and SARS-CoV-2 infection. His lisinopril was suspended on admission. By day 3 of admission, his swelling significantly improved, and the intravenous Solu-Medrol, diphenhydramine, and remdesivir were discontinued. He had resolution of his shortness of breath, dry cough, and body aches. The patient was discharged on day 4 of admission with amlodipine rather than lisinopril for blood pressure management.

## Discussion

Angioedema is a condition characterized by asymmetric, non-pitting edema of the subcutaneous or submucosal tissues particularly in the face, lips, and oral cavity [1]. ACE inhibitors are a medication class that is associated with angioedema in 0.1-0.7% of treated patients [5]. ACE inhibitor-induced angioedema occurs more frequently in African Americans, females, smokers, seniors, and those with seasonal allergies [3,6,7]. Most patients experience ACE inhibitor-induced angioedema a few weeks to a few months after starting an ACE inhibitor [8]. The underlying pathophysiology is secondary to ACE inhibitors binding to ACE, leading to a decrease in the formation of angiotensin II and preventing the breakdown of bradykinin into inactive metabolites. Heightened levels of bradykinin and its active metabolites lead to plasma extravasation and edema formation [9]. The diagnosis of ACE-induced angioedema is based on the presence of angioedema without urticaria or pruritis in a patient taking ACE inhibitors. There are no routine laboratory tests to confirm the diagnosis. In our patient, hereditary angioedema was unlikely given the use of ACE inhibitors prior to admission, lack of personal or family history of angioedema, and a C4 level of 44 mg/dL. The gradual onset of swelling, no urticarial lesions on physical examination, and history negative for recent exposure to allergic triggers suggested histamine-mediated angioedema was doubtful.

In the case of suspected angioedema, this class of drug should be immediately halted. After discontinuation of the ACE inhibitor, edema usually resolves on its own within 48-72 hours [8]. Patients with angioedema localized to the face, lips, and throat should undergo further evaluation by an ENT physician, potentially with the aid of laryngoscopy to assess the patency of the upper respiratory tract. There is no standardized approach for the treatment of ACE inhibitor-induced angioedema. Administering systemic glucocorticoids and antihistamines is generally of limited efficacy [10]. Epinephrine is indicated in severe cases of angioedema. C1 inhibitor concentrate may be of benefit by downregulating bradykinin; however, the results are non-conclusive [10]. The bradykinin receptor antagonist icatibant has shown conflicting efficacy in randomized controlled trials [11,12]. Patients who have shown a clear episode of ACE inhibitor-induced angioedema are recommended to be switched to a different class of anti-hypertensives to prevent the recurrence of angioedema attacks.

SARS-CoV-2 can result in extensive systemic inflammation, endothelial dysfunction, and increased vascular permeability [4]. Roche and Roche hypothesize that the virus impairs the breakdown of bradykinin leading to lung injury, inflammation, and respiratory complications [13]. SARS-CoV-2 is believed to contribute to the development of angioedema via a "second-hit" phenomenon. The virus, by binding to the ACE II receptor, depletes ACE II, thereby leading to an increase in the des-Arg9-bradykinin level, an active bradykinin metabolite [14]. There have been a few case reports of angioedema occurring in patients with concomitant ACE inhibitor use and SARS-CoV-2 infection [15-20]. This case strengthens a potential interplay between these two factors in the development of angioedema. There are no guidelines on the management of patients with ACE inhibitor-induced angioedema and a positive SARS-CoV-2 result. Management approach in previous cases consisted of cessation of the ACE inhibitor in all cases. In four cases, systemic glucocorticoids and anti-histamines were initiated [15,17,20]. In two cases, only systemic glucocorticoids were implemented [18,19]. In one case, tranexamic acid was used with clinical improvement [16]. Our patient was treated with systemic glucocorticoids, anti-histamines, and remdesivir with resolution of angioedema on day 3 of admission. Systemic glucocorticoids and anti-histamines were used as histamine-mediated angioedema could not be definitively ruled out. Further research is needed to characterize the effect of SARS-CoV-2 on bradykinin receptor signaling pathways and its role in the development of angioedema in patients taking ACE inhibitors. Also, given the prevalence of ACE inhibitors for the treatment of hypertension, recommendations on the management of ACE inhibitor-induced angioedema in patients with SARS-CoV-2 infection would be beneficial.

In the United States, African Americans infected with SARS-CoV-2 have a disproportionately higher morbidity and mortality as compared to other ethnic groups [21]. African Americans are known to be at an increased risk of angioedema due to an increased sensitivity to bradykinin and polymorphisms in the genes encoding neutral endopeptidase (NEP) and aminopeptidase P (APP) [22,23]. These polymorphisms are associated with decreased levels of circulating enzymes involved in the breakdown of bradykinin and des-Arg9-bradykinin [22]. Therefore, alterations in bradykinin receptor signaling may play a role in this ethnic group having a predisposition to angioedema and other respiratory complications when infected with SARS-CoV-2. Batarseh et al. reported four cases of angioedema in African American patients hospitalized with SARS-CoV-2 infection [19]. Out of the four cases, one patient was taking lisinopril prior to admission. More studies are needed to investigate the risk of angioedema and other respiratory complications in African Americans infected with SARS-CoV-2 with and without the presence of an ACE inhibitor.

## Conclusions

ACE inhibitor-induced angioedema is a rare complication in patients receiving ACE inhibitors. Infection with SARS-CoV-2 may give rise to a synergistic effect that leads to angioedema in patients receiving ACE inhibitors. African Americans could be at particular risk of developing this "second hit" secondary to a genetic predisposition to developing angioedema. There is a need to further understand the interplay between ACE inhibitors and SARS-CoV-2 in the development of angioedema, especially in the African American population.

## References

[1] Banerji A, Clark S, Blanda M, LoVecchio F, Snyder B, Camargo CA Jr (2008). Multicenter study of patients with angiotensin-converting enzyme inhibitor-induced angioedema who present to the emergency department. Ann Allergy Asthma Immunol.

[2] Messerli FH, Nussberger J (2000). Vasopeptidase inhibition and angio-oedema. Lancet.

[3] Miller DR, Oliveria SA, Berlowitz DR, Fincke BG, Stang P, Lillienfeld DE (2008). Angioedema incidence in US veterans initiating angiotensin-converting enzyme inhibitors. Hypertension.

[4] Guzik TJ, Mohiddin SA, Dimarco A (2020). COVID-19 and the cardiovascular system: implications for risk assessment, diagnosis, and treatment options. Cardiovasc Res.

[5] Kostis WJ, Shetty M, Chowdhury YS, Kostis JB (2018). ACE inhibitor-induced angioedema: a review. Curr Hypertens Rep.

[6] Kostis JB, Kim HJ, Rusnak J, Casale T, Kaplan A, Corren J, Levy E (2005). Incidence and characteristics of angioedema associated with enalapril. Arch Intern Med.

[7] Loftus PA, Tan M, Patel G (2014). Risk factors associated with severe and recurrent angioedema: an epidemic linked to ACE-inhibitors. Laryngoscope.

[8] Montinaro V, Cicardi M (2020). ACE inhibitor-mediated angioedema. Int Immunopharmacol.

[9] Nussberger J, Cugno M, Cicardi M (2002). Bradykinin-mediated angioedema. N Engl J Med.

[10] Moellman JJ, Bernstein JA, Lindsell C (2014). A consensus parameter for the evaluation and management of angioedema in the emergency department. Acad Emerg Med.

[11] Baş M, Greve J, Stelter K (2015). A randomized trial of icatibant in ACE-inhibitor-induced angioedema. N Engl J Med.

[12] Sinert R, Levy P, Bernstein JA (2017). Randomized trial of icatibant for angiotensin-converting enzyme inhibitor-induced upper airway angioedema. J Allergy Clin Immunol Pract.

[13] Roche JA, Roche R (2020). A hypothesized role for dysregulated bradykinin signaling in COVID-19 respiratory complications. FASEB J.

[14] Rostamzadeh F, Najafipour H, Nakhaei S, Yazdani R, Langari AA (2023). Low Ang-(1-7) and high des-Arg9 bradykinin serum levels are correlated with cardiovascular risk factors in patients with COVID-19. Open Med (Wars).

[15] Sabalenka TM, Zakharava VV, Prakoshyna NR (2021). Angioedema induced by angiotensin-converting enzyme inhibitors: an analysis of hospitalizations during the COVID-19 pandemic. Russian Journal of Allergy.

[16] Grewal E, Sutarjono B, Mohammed I (2020). Angioedema, ACE inhibitor and COVID-19. BMJ Case Rep.

[17] Kuzemczak M, Kavvouras C, Alkhalil M, Osten M (2021). ACE inhibitor-related angioedema in a COVID-19 patient-a plausible contribution of the viral infection?. Eur J Clin Pharmacol.

[18] Monteleone G, Maria Leone P, Bonini M, Corbo G, Leone A (2023). Were SARS-Cov-2 or ACE-I responsible for angioedema or was it caused by a second strike? A case report. Euromediterranean Biomedical Journal.

[19] Batarseh E, Kersten BP, Pinelo AC, Nadler JN, Schwartz SA (2020). Angioedema in African American patients hospitalized for COVID-19. Am J Respir Crit Care Med.

[20] Cohen AJ, DiFrancesco MF, Solomon SD, Vaduganathan M (2020). Angioedema in COVID-19. Eur Heart J.

[21] Yancy CW (2020). COVID-19 and African Americans. JAMA.

[22] Campo P, Fernandez TD, Canto G, Mayorga C (2013). Angioedema induced by angiotensin-converting enzyme inhibitors. Curr Opin Allergy Clin Immunol.

[23] Woodard-Grice AV, Lucisano AC, Byrd JB, Stone ER, Simmons WH, Brown NJ (2010). Sex-dependent and race-dependent association of XPNPEP2 C-2399A polymorphism with angiotensin-converting enzyme inhibitor-associated angioedema. Pharmacogenet Genomics.

